# *Thelazia callipaeda* in mustelids from Romania with the European badger, *Meles meles*, as a new host for this parasite

**DOI:** 10.1186/s13071-019-3631-4

**Published:** 2019-07-26

**Authors:** Angela Monica Ionică, Georgiana Deak, Gianluca D’Amico, Gheorghe Florin Stan, Gabriel Bogdan Chișamera, Ioana Cristina Constantinescu, Costică Adam, Menelaos Lefkaditis, Călin Mircea Gherman, Andrei Daniel Mihalca

**Affiliations:** 1 Department of Parasitology and Parasitic Diseases, University of Agricultural Sciences and Veterinary Medicine Cluj-Napoca, 3-5 Calea Mănăştur, 400372 Cluj-Napoca, Romania; 2 CDS-9, “Regele Mihai I al României” Life Science Institute, University of Agricultural Sciences and Veterinary Medicine Cluj-Napoca, 3-5 Calea Mănăştur, 400372 Cluj-Napoca, Romania; 3 Department of Anatomy, University of Agricultural Sciences and Veterinary Medicine Cluj-Napoca, 3-5 Calea Mănăştur, 400372 Cluj-Napoca, Romania; 4 “Grigore Antipa” National Museum of Natural History, Sos. Kiseleff no. 1, 011341 Bucharest 1, Romania; 5 Laboratory of Microbiology and Parasitology, Faculty of Veterinary Medicine, School of Health Sciences, University of Thessaly, 224 Trikalon, Karditsa, Greece

**Keywords:** *Thelazia callipaeda*, Host, Mustelidae, *Meles meles*, *Martes foina*

## Abstract

**Background:**

*Thelazia callipaeda* (Spirurida, Thelaziidae) is a vector-borne zoonotic eye worm with a broad host spectrum. In Europe, it is an emerging threat, having greatly expanded its geographical distribution during the past two decades. In Romania, *T*. *callipaeda* has been previously reported in domestic and wild canids and felids. The aim of the present study was to assess the occurrence of *T. callipaeda* in mustelids in the country.

**Methods:**

Between March 2015 and April 2019, 77 road-killed mustelids (3 pine martens, *Martes martes*; 6 European polecats, *Mustela putorius*; 13 beech martens, *Martes foina*; and 55 European badgers, *Meles meles*) were examined by necropsy. If present, all ocular nematodes were collected and stored in absolute ethanol, for subsequent morphological and molecular identification.

**Results:**

Two animals were found to be infected with *T. callipaeda*: one European badger and one beech marten. The molecular analysis revealed a 100% nucleotide similarity to *T. callipaeda* haplotype h1 for all the sequenced specimens.

**Conclusions:**

To our knowledge, the present study demonstrates for the first time the occurrence of *T. callipaeda* in mustelids from Romania, records the easternmost locality of the parasite in Europe, and represents the first report of *T. callipaeda* in the European badger, *Meles meles*, extending the known host range for this parasite in Europe.

## Background

*Thelazia callipaeda* (Spirurida: Thelaziidae) is a vector-borne zoonotic nematode residing in the conjunctival sac of a variety of hosts, including domestic and wild carnivores, lagomorphs and humans [1, 2]. Its occurrence is associated with variable clinical presentations, ranging from asymptomatic carriage to mild (discharge, epiphora, conjunctivitis) or severe (keratitis, ulcers) ocular disease [3, 4]. In Europe, the only confirmed vectors for *T. callipaeda* are male *Phortica variegata* (Drosophilidae: Steganinae), which deposit infective L3 larvae while feeding on ocular secretions of the receptive hosts [5].

Considering the origin of this parasite in Far Eastern countries, it has been for a long time referred to as the “oriental eye worm” [6]. *Thelazia callipaeda* is regarded as an emerging zoonotic agent in Europe, with the list of endemic countries having greatly expanded during the past two decades, currently including Austria, Bosnia and Herzegovina, Bulgaria, Croatia, France, Germany, Greece, Hungary, Italy, Portugal, Romania, Serbia, Slovakia, Spain, Switzerland and Turkey [7–10]. Additionally, human cases have also been documented in some endemic regions (Croatia, France, Italy, Serbia and Spain), underlining the relevance of this parasite to public health [7]. However, most reports have focused on domestic carnivores, while data regarding wildlife is still scarce.

In Romania, *T. callipaeda* infection was documented in domestic dogs [11–13] and domestic cats [14]. Subsequent research targeting wild canids and felids also revealed the occurrence of the parasite in gray wolves (*Canis lupus*), golden jackals (*Canis aureus*), wildcats (*Felis silvestris*) [15] and red foxes (*Vulpes vulpes*) [16], pointing out a wide host spectrum of the parasite. However, the role of other carnivores as wild reservoirs for *T. callipaeda* has been poorly investigated. Among these, mustelids are abundant but due to their elusive lifestyle, small size and, in many cases, protected status, studies on the role of these species in the natural cycle of *T. callipaeda* are limited [1]. Considering the recent emergence and almost countrywide distribution of this parasite in Romania, the aim of the present study was to investigate the occurrence of *T. callipaeda* in mustelid species from Romania.

## Methods

Between March 2015 and April 2019, 77 road-killed and hunted mustelids originating from 15 counties from western, north-western, central, southern and eastern Romania were examined by necropsy: 55 European badgers, *Meles meles*; 13 beech martens, *Martes foina*; 3 pine martens, *Martes martes*; and 6 European polecats, *Mustela putorius* (Table 1). During the necropsy for each animal, both eyes were thoroughly checked for the presence of parasites including *Dirofilaria repens*, *Onchocerca lupi* and *Thelazia callipaeda*. If present, all nematodes were collected and stored in absolute ethanol, for subsequent morphological and molecular identification.

**Table 1:** The distribution of examined animals according to region and year

Year	Western	North-western	Central	Southern	Eastern
2015	0	*Meles meles* (*n* = 1)	*Meles meles* (*n* = 1)	0	*Martes foina* (*n* = 1)
2016	*Meles meles* (*n* = 1)^a^; *Martes foina* (*n* = 1); *Mustela putorius* (*n* = 2)	*Meles meles* (*n* = 9); *Martes foina* (*n* = 3); *Martes martes* (*n* = 1)	*Meles meles* (*n* = 2)	0	0
2017	0	*Meles meles* (*n* = 9)	*Meles meles* (*n* = 4); *Martes foina* (*n* = 1); *Martes martes* (*n* = 1); *Mustela putorius* (*n* = 1)	*Mustela putorius* (*n* = 1)	0
2018	0	*Meles meles* (*n* = 19); *Martes foina* (*n* = 5); *Martes martes* (*n* = 1)	*Meles meles* (*n* = 6); *Mustela putorius* (*n* = 1)	*Martes foina* (*n* = 1); *Mustela putorius* (*n* = 1)	0
2019	0	*Meles meles* (*n* = 3)	0	0	*Martes foina* (*n* = 1)^a^

^a^Positive for *T. callipaeda* infection

All of the collected nematodes were temporarily mounted on glass slides, cleared with lactophenol and examined under a light microscope (Olympus BX61, Olympus Corporation, Tokyo, Japan). The morphological identification was performed based on keys and descriptions from literature [17]. Subsequently, the nematodes were washed with ethanol and stored in labeled cryotubes.

In order to confirm the haplotype, at least one specimen from each positive animal was genetically characterized. Briefly, genomic DNA was isolated using a commercial kit (Isolate II Genomic DNA Kit, Bioline, London, UK) and the samples were processed by PCR amplification and sequencing of a 670-bp fragment of the *cox*1 gene using the NTF/NTR primer pair, as previously described [18].

## Results

**Fig. 1:**
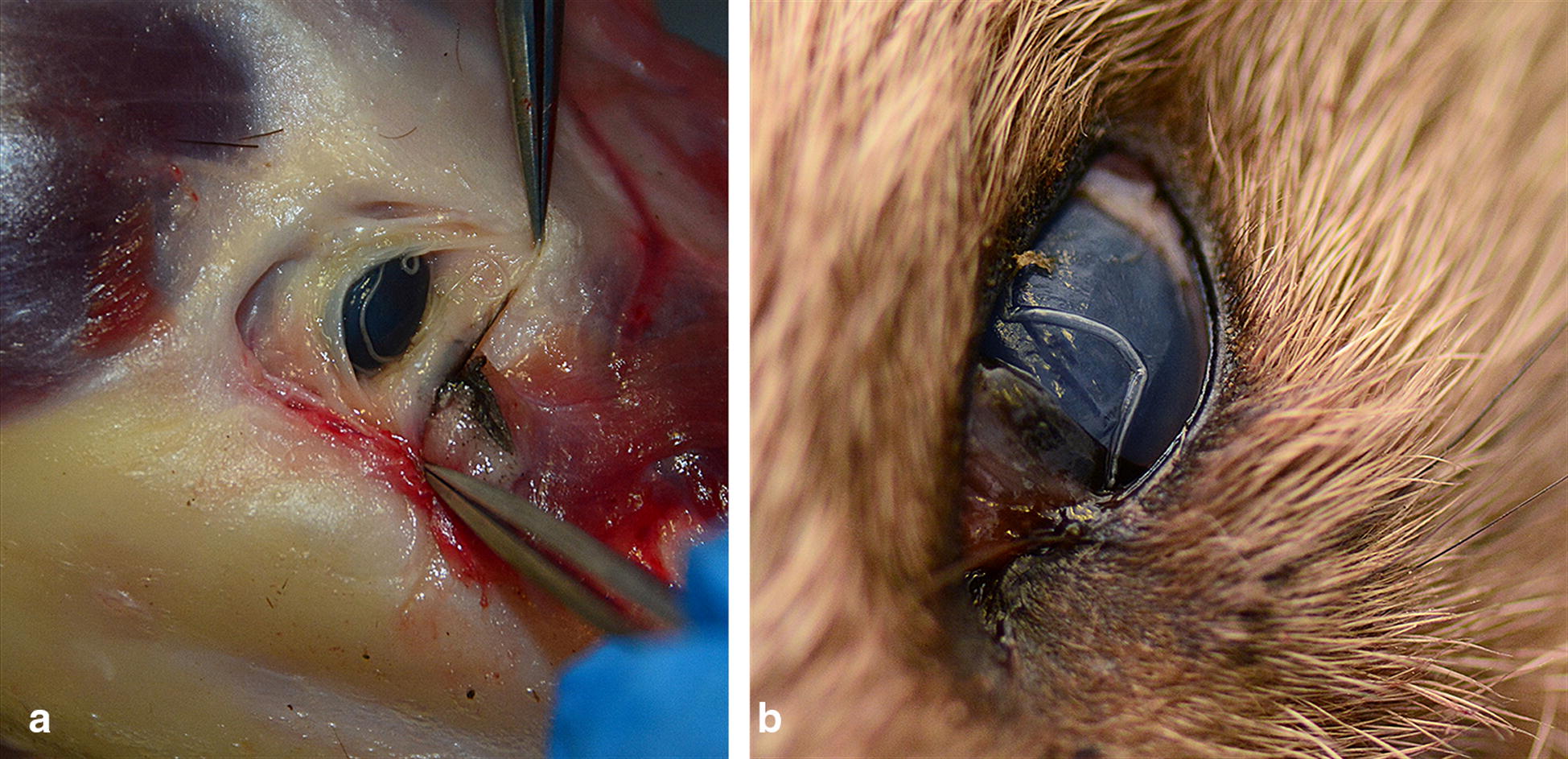
*Thelazia callipaeda* in mustelids from Romania in the conjunctival sac of a European badger, *Meles meles* (**a**), and on the cornea of a beech marten, *Martes foina* (**b**)

**Fig. 2:**
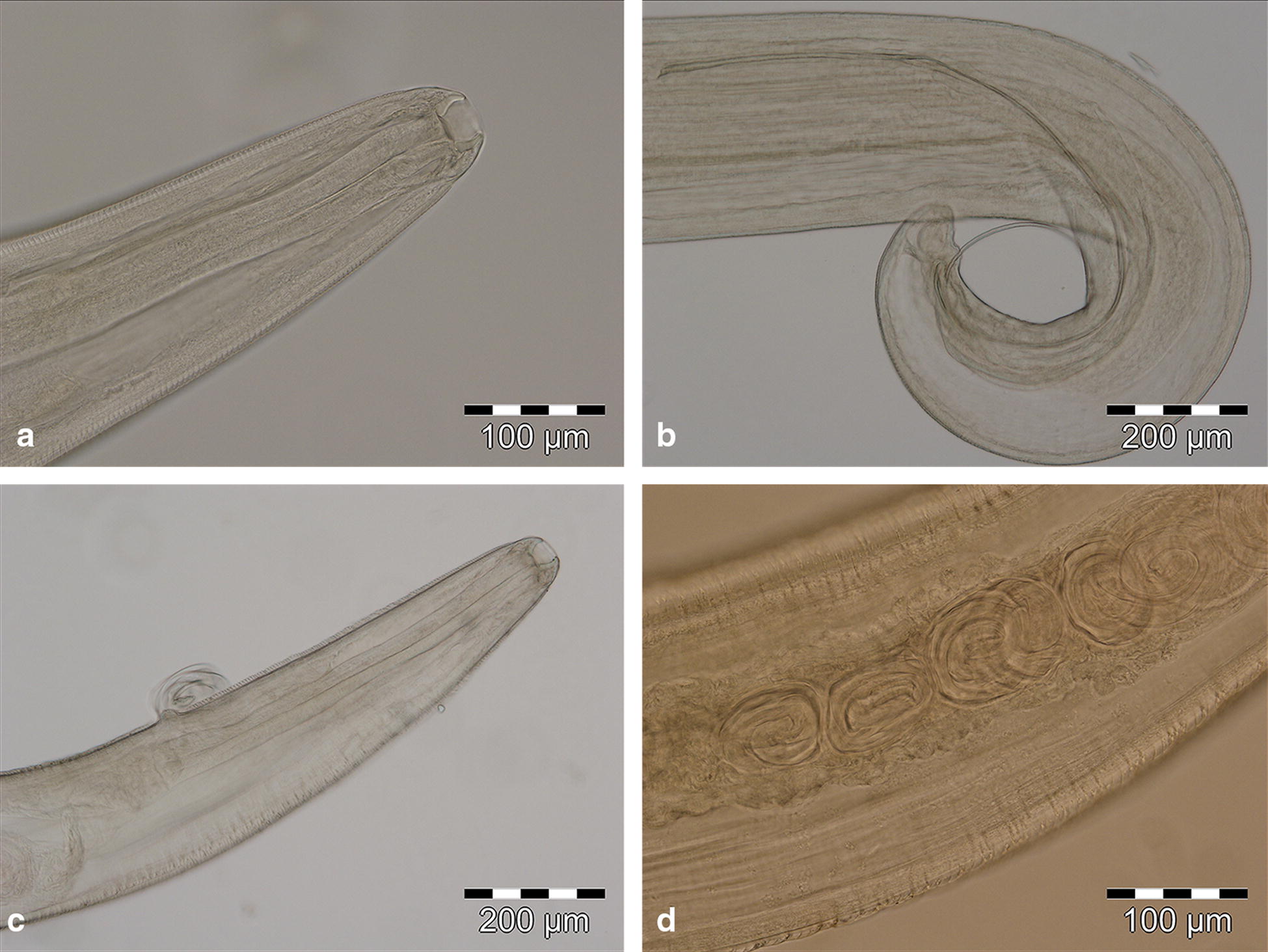
*Thelazia callipaeda* nematodes recovered from the European badger, *Meles meles*. **a** Anterior extremity, buccal capsule and mouth opening with a hexagonal profile, transversally serrated cuticle. **b** Posterior extremity of male. **c** Gravid female, vulva located anterior to the oesophago-intestinal junction. **d** Gravid female, larvae inside the uterus

Among all examined mustelids, two animals were positive for eye worms (Fig. 1). The first was an adult female European badger, *Meles meles* (1.82%; 95% CI: 0.05–9.72%), originating from the western part of the country (45°58′32.97′′N, 21°31′7.546′′E). Overall, 33 nematodes identified as *T. callipaeda* were recovered: 23 males and 10 gravid females (Fig. 2). The animal harbored bilateral infection, with 10 specimens present at the left eye level (7 males and 3 females) and the rest (16 males and 7 females) at the right eye level. Two randomly chosen specimens (one male and one female) were sequenced.

The second positive mustelid was an adult male beech marten, *Martes foina* (7.69%; 95% CI: 0.19–36.03%), originating from eastern Romania (45°10′37.564′′N, 28°36′22.406′′E). A single female nematode was recovered from the right eye and also sequenced.

The three attained sequences were identical to each other and showed a 100% similarity to a sequence of *T. callipaeda* haplotype h1 (GenBank: AM042549) by BLAST analysis.

## Discussion

To date, among mustelids in Europe, *T. callipaeda* infection has been reported only in beech martens, *Martes foina*: three animals (13.6%) from southern Italy [1] and one case report from north-central Portugal [19]. To the best of our knowledge, our results represent the first report of *T. callipaeda* infection in the European badger, *Meles meles*. A previous study performed in a hyperendemic area of Italy included ten badgers, but they were all negative; this was attributed to the almost complete lack of diurnal activity of this species [1]. Indeed, a behavioral study has shown that during spring and autumn, badgers start their activity around one hour after sunset and end it around two hours before sunrise. However, during the summer, they emerged around one hour before the sunset and returned to the sett around half hour after sunrise [20]. Therefore, at least during the summer, contact between the badgers and the vectors, which display crepuscular activity, is possible [21]. According to observed movement patterns, which involve various degrees of diurnal activity [22–24], martens and polecats would theoretically be more exposed to the vectors compared to badgers. Indeed, the relative frequency in beech martens seems to be higher compared to badgers. However, negativity of pine martens and polecats may be a consequence of the small sample size and their role as hosts for *T. callipaeda* still remains to be clarified.

**Fig. 3:**
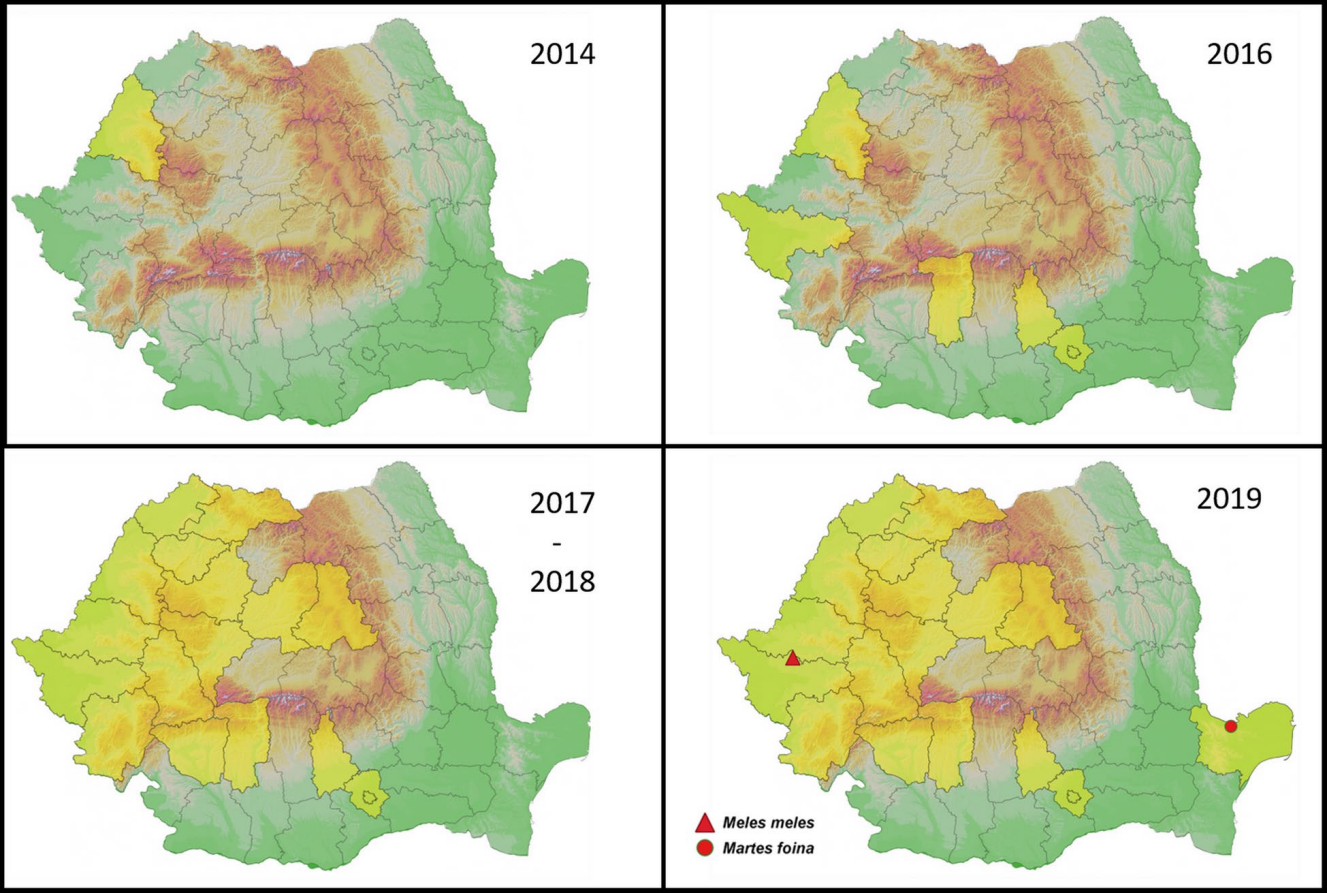
The emergence of the known distribution of *Thelazia callipaeda* in Romania. Positive counties are highlighted in yellow (based on data in [11–16]). The location of the positive mustelids is marked accordingly

The infected badger originated from western Romania, an area where the vector has been previously recorded [11] and the occurrence of the parasite in domestic dogs and red foxes has also been demonstrated [12, 16]. Interestingly, the positive marten was found in the eastern part of the country, representing, to our knowledge, the first record of the parasite in the area (Fig. 3) and the easternmost location in Europe so far. The presence of the vector has never been reported or investigated in the region, but according to the predictive distribution model, it would be environmentally suitable for the presence of *P. variegata* [7].

Considering the activity pattern of mustelids in general and the subterranean lodging of badgers while inactive in particular, they probably do not represent a major reservoir. However, the present cases indicate that they can be suitable hosts for *T. callipaeda*, having a potential involvement in the parasite’s sylvatic life-cycle.

## Conclusions

The present study demonstrates the occurrence of *T. callipaeda* infection in mustelids from Romania. Furthermore, to our knowledge, it represents the first report of *T. callipaeda* in the European badger, *Meles meles*, and the easternmost record of the parasite in Europe extending the known geographical and host range for this parasite.

## Data Availability

Data supporting the conclusions of this article are included within the article. The newly generated sequences were submitted to the GanBank database under the accession numbers MN176281–MN176282. The raw datasets used and/or analysed during the present study are available upon request.
